# The effect of noise on the amplitude and morphology of cortical auditory evoked potentials

**DOI:** 10.1016/j.bjorl.2021.11.006

**Published:** 2021-12-07

**Authors:** Danielle Samara Bandeira Duarte, Silvana Maria Sobral Griz, Mônyka Ferreira Borges Rocha, Diana Babini Lapa de Albuquerque Britto, Denise Costa Menezes, Karina Paes Advíncula

**Affiliations:** a Universidade Federal de Pernambuco (UFPE), Recife, PE, Brazil; b Universidade Federal de Pernambuco (UFPE), Departamento de Fonoaudiologia e Programa de Pós-Graduação em Saúde da Comunicação Humana, Recife, PE, Brazil; c Universidade Federal de Alagoas (UFAL), Maceió, AL, Brazil; d Universidade Federal de Pernambuco (UFPE), Departamento de Fonoaudiologia, Recife, PE, Brazil

**Keywords:** Electrophysiology, Auditory evoked potentials, Speech perception, Aging, Hearing

## Abstract

**Objective:**

To analyze the effect of noise on electrophysiological measurements (P1-N1-P2 complex) of cortical auditory evoked potentials in normal hearing individuals of different ages.

**Methods:**

The inclusion criteria for the study were young individuals, adults and elderly, aged 18–75 years, with auditory thresholds up to 25 dB. Participants were separated according to their age group: G1 (18–25 years old), G2 (31–59 years old) and G3 (60–75 years old). Cortical auditory evoked potentials were elicited with synthetic speech stimulus /da/ presented in two conditions: without masking and with masking (Delta-t 64 ms). The results were expressed and analyzed using statistical measures.

**Results:**

High latencies and reduced amplitudes were observed in the Delta-t 64 ms condition, in all age groups. There were significant differences between the groups, both in P1 latencies for the two conditions and in N1 latencies in the Delta-t 64 ms condition. P1 latencies in the condition without masking were lower in G1 and P1 and N1 latencies in the Delta-t 64 ms condition were higher in G3. The described results show the influence of noise on cortical responses in all age groups, with G3 being the most affected by the masking presentation.

**Conclusion:**

The latency and amplitude measurements vary according to the stimulus presentation condition and age group. The forward masking phenomenon occurred with greater precision in G3.

**Level of evidence:**

(2c).

## Highlights

• The presence of masking noise interferes with latency and amplitude measurements of Cortical Auditory Evoked Potentials.
• Presence of forward masking phenomenon during the measurements in the masking noise presentation condition before the speech stimulus.
• Greater presence of forward masking phenomenon in the elderly group.

## Introduction

The integration of the Peripheral Auditory System (PAS) and the Central Auditory Nervous System (CANS) makes it possible to detect, discriminate, recognize and understand sound stimuli. For speech recognition, the systems receive combinations of cues, such as: intensity, phoneme frequency range, prosody, familiarity with acoustic signal timing and linguistic context.^1^, ^2^

To successfully process these cues, the auditory system must be able to focus attention on the speech signal only, ignoring the noisy signals from the environment. Competitive noise is very common in the everyday lives of listeners and can make it difficult to understand the speech during a conversation.^3^

In the presence of different types of noise, speech detection thresholds can be increased. The modulation in noise intensity and frequency helps in speech understanding, due to the increase in the signal-to-noise ratio caused by the reduction in masking intensity levels at the time of minimum modulation. Therefore, the individual starts to hear the target sound exactly at the moment of minimum modulation, when the masking noise is at its weakest intensity, obtaining enough information to perform target sound detection and decoding. This phenomenon is called Modulation Masking Release (MMR).^4^, ^5^

With advancing age, individuals can progressively lose the ability to detect speech in the presence of competitive noise (even in the presence of modulated noise). That is, the elderly have lower MMR when compared to younger listeners.^5^, ^6^

Several factors must be considered for the reduction of MMR in senescence, such as: decreased audibility, reduced processing of speech redundancy and reduced temporal masking. Even with auditory thresholds considered within the normal range,^7^ elderly individuals have difficulty in detecting the speech signal in the presence of modulated noise. This failure is thought to be caused by one of the characteristics of temporal masking, more specifically, the forward masking phenomenon.^5^

Temporal masking is understood as the change in the sound detection threshold caused by the presence of another sound (masking noise). The noise can be presented before (forward masking), after (backward masking) or concomitantly with the target sound (simultaneous masking). The presentation of noise prior to the target sound triggers the forward masking phenomenon, characterized by the interference of the masking sound in the target sound detection, even after its physical presence is reduced or ended. This phenomenon is elicited through specific methodologies during the performance of long latency auditory evoked potentials, also called Cortical Auditory Evoked Potentials (CAEPs).^5^ The hypothesis is that this phenomenon occurs because the hair cells, previously stimulated by the masking noise, have not fully recovered their resting sensitivity. Thus, the processing of the target sound, presented after the masking sound, may be impaired.^6^

The reduced MMR in the elderly can occur due to the increase in the magnitude of the temporal masking, decreasing the capacity of the elderly auditory system to capture the acoustic cues in the temporal spaces when there is a reduction in the intensity of modulated noise, when compared to younger listeners.^5^

The cortical auditory evoked potentials are efficient electrophysiological responses in the analysis of neurophysiological changes in the central auditory nervous system, being used in MMR screening and can be applied in the post-masking investigation. Bioelectrical activities from acoustic stimulation in the thalamocortical regions generate a P1-N1-P2 wave complex, allowing the study of the central auditory function. These potentials are considered to be exogenous, that is, they do not need the individual's attention to be elicited.^8^, ^9^, ^10^

Considering the importance of further studies on post-masking in electrophysiological measurements, this study allows us to understand this phenomenon for auditory cortical processes in senescence. Moreover, it may contribute to the measurement of electrophysiological markers for the normal hearing population,^7^ in different age groups, which can be used as the basis for further research involving CANS alterations.

This study aimed to analyze the effect of noise on electrophysiological measurements (P1-N1-P2 complex) of CAEPs in normal-hearing young adults, adults and the elderly.

## Methods

This is an analytical, observational and cross-sectional study, approved by the Research Ethics Committee, under Opinion number 3,555,712.

The inclusion criteria for the research comprised: young adult (18–30 years old), adult (31–59 years old) and elderly (60–75 years old) participants, with hearing thresholds in the frequencies of 500 Hz, 1000 Hz and 2000 Hz, lower or equal at 25 dB HL,^7^ excluding individuals with a history of neurological and/or psychiatric diseases, cognitive deficits, individuals with inner ear alterations, malformations of the ear pinna and external auditory canal that would make it impossible to perform the CAEP exam.

The individuals were divided into three groups: G1 (10 young adults), G2 (10 adults) and G3 (10 elderly individuals). All participants received information regarding the collection objectives and procedures and signed the Free and Informed Consent Form (FICF) in duplicate, after agreeing to participate in the study. Next, the pre-collection exams were scheduled to meet the research eligibility criteria. Detailed audiological history, basic audiological examinations (external auditory canal inspection, tonal and vocal audiometry, and immitance audiometry) and the Montreal Cognitive Assessment test – MoCA were performed.^11^

Alterations in the external and/or middle ear were investigated through the immitance audiometry test, being considered normal in the presence of a type A tympanometry curve and the presence of ipsilateral and contralateral reflexes.^12^, ^13^ In the pure tone audiometry exam, the thresholds were obtained for the frequencies between 250 Hz and 8000 Hz, including 3000 Hz and 6000 Hz, in both ears. In the MoCA test, a score equal to or greater than 26 points, described in the test, was considered a normal result.^11^

For the CAEP examination, the Speech Shaped Noise (SSN) was used, with an intensity of 80 dBHL and 365 ms in duration. The synthetic speech stimulus /da/ was used because it is a universal syllable and elicits clearer and more replicable responses.^14^ The stimulus was presented with an intensity of 75 dB SPL with a loading time of 10 ms, 59.05 ms of plateau duration and a recovery time of 10 ms. The acquisition parameters used were: 1 Hz high-pass filter and 30 Hz low-pass filter; 800 ms window, minimum of 100 presented stimuli and two trace reproductions; and a presentation rate of 0.7 s. The noise was created in the Laboratory of Hearing Sciences of University of North Carolina at Chapel Hill, USA, and the speech stimulus /da/ used in the study was created by Nina Kraus.

The individuals underwent the CAEP test utilizing the Intelligent Hearing Systems – IHS, model Opti-Amp 8008 equipment. The speech stimulus /da/ and the masking noise were presented monaurally in the right ear, through an insertion earphone (ER3).

To perform the exam, the participant was positioned in a reclining chair inside an acoustically treated booth, concentrating on some abstract activity to divert their attention from the auditory stimulus. The participants were instructed not to sleep during the exam. The skin was cleaned with 70% alcohol and NuPrep® abrasive gel. Subsequently, electrodes were applied according to the following configurations: two reference electrodes with negative polarity were placed on the region of the right (A2) and left (A1) earlobes; a positive polarity electrode was placed at the vertex (Cz) and the ground electrode was placed at the lower frontal region (Fpz). The impedance was ≤5 kΩ.

The forward masking phenomenon analysis was carried out under two conditions: (1) CAEP with speech stimulus /da/ without the presence of masking noise (without masking) and (2) CAEP with masking noise presented 64 ms before the speech stimulus /da/ (Delta-t 64 ms). To capture the Delta-t 64 ms condition, the noise was presented at time 0 ms, with a duration of 365 ms, an interval of 64 ms until the presentation of the synthetic speech stimulus at around 429 ms. The objective of this step was to evaluate the presence or absence of forward masking phenomenon using the measurement of the P1-N1-P2 complex response of the CAEP.

Latencies (in milliseconds – ms) and amplitudes (in microvolts – µV), as well as the morphology of the P1, N1, P2 waves were analyzed for both conditions. In the condition without masking, the P1 component was considered the most robust first positive cortical wave around 50 ms and in the Delta-t 64 ms condition, the P1 component was observed around 250 ms, considering the masking noise presentation time between −100 ms and 200 ms. Wave N1 was analyzed as the valley subsequent to wave P1, with greater negativity. P2 was identified as the most robust positive wave after wave N1.

The results were descriptively analyzed through frequencies in categorical variables and through means with standard deviation (mean ± SD), median and 25th and 75th percentiles (median (median (P25; P75)) for numerical variables. For the comparison of latencies between conditions without masking and Delta-t 64 ms, Student’s paired *t* test or Wilcoxon paired test and F (ANOVA) or Kruskal–Wallis tests were used in the comparison between age groups. In case of significant differences, in the F (ANOVA) test, Tukey or Tamhane multiple comparison tests (between pairs) were used. When the difference was recorded by the Kruskal–Wallis test, Conover comparisons were performed. The choice of the paired Student’s *t* test and F (ANOVA) occurred in the situations in which the data showed normal distribution and the Wilcoxon paired and Kruskal–Wallis tests were used in the case of rejection of normality. The choice of Tukey comparisons occurred when an equality of variance was verified, and Tamhane was chosen when there was rejection of the equality of variances. The verification of normality and equality of variances were respectively performed by Shapiro–Wilk and Levene’s F tests.

The margin of error used in the choice of the statistical tests was 5%. Data were entered into an Excel spreadsheet and the programs used to perform the statistical calculations were IMB SPSS, version 25.0 and MEDCALC version 19.2.6.

## Results

Of the total number of participants, 10 were from the group of young adults aged between 20 and 26 years, 10 from the group of adults aged from 35 to 38 years and 10 from the elderly group, aged 61–75 years. Twenty-five of the participants were females (63.3%) and 5 were males (16.7%).

Table 1 shows that, although all participants had their hearing thresholds in the frequencies of 500 Hz, 1000 Hz and 2000 Hz within the normal range (up to 25 dBHL),^7^ the elderly group had higher thresholds when compared to the other groups.

**Table 1:** Description of the mean, standard deviation and median of the participants' hearing thresholds.

Frequency	Young adult group	Adult group	Elderly group	*p* Value
	Mean ± SD	Mean ± SD	Mean ± SD	
	Median (P25; P75)	Median (P25; P75)	Median (P25; P75)	
250 Hz	17.50 ± 4.25	17.00 ± 2.58	22.50 ± 3.54	*p*^a^ = 0.006^b^
	17.50 (15.00; 20.00)	15.00 (15.00; 20.00)	25.00 (20.00; 25.00)	
500 Hz	18.00 ± 4.22	16.50 ± 4.74	21.50 ± 3.37	*p*^a^ = 0.039^b^
	20.00 (15.00; 20.00)	15.00 (13.75; 20.00)	20.00 (20.00; 25.00)	
1000 Hz	18.50 ± 4.12	16.00 ± 3.94	19.50 ± 3.69	*p*^a^ = 0.161
	20.00 (15.00; 20.00)	15.00 (13.75; 20.00)	20.00 (15.00; 21.25)	
2000 Hz	16.50 ± 2.42	16.00 ± 4.59	19.50 ± 4.97	*p*^a^ = 0.166
	15.00 (15.00; 20.00)	17.50 (10.00; 20.00)	20.00 (15.00; 25.00)	
3000 Hz	19.50 ± 1.58	18.00 ± 2.58	21.00 ± 3.94	*p*^a^ = 0.101
	20.00 (20.00; 20.00)	20.00 (15.00; 20.00)	20.00 (18.75; 25.00)	
4000 Hz	17.50 ± 2.64	18.50 ± 4.12	23.50 ± 2.42	*p*^a^ = 0.001^b^
	17.50 (15.00; 20.00)	20.00 (15.00; 20.00)	25.00 (20.00; 25.00)	
6000 Hz	16.00 ± 3.94	17.00 ± 5.37	30.00 ± 12.02	*p*^a^ = 0.002^b^
	15.00 (13.75; 20.00)	15.00 (13.75; 21.25)	25.00 (23.75; 37.50)	
8000 Hz	15.50 ± 3.69 ^(A)^	16.00 ± 3.94 ^(A)^	37.50 ± 18.45 ^(B)^	*p*^a^ < 0.001^b^
	15.00 (13.75; 20.00)	15.00 (13.75; 20.00)	30.00 (20.00; 60.00)	

SD, standard deviation.

a Kruskal Wallis test with Conover’s all-pairs comparison.

b Significant difference at the 5.0% level.

Table 2, Table 3 show the results of latencies and amplitudes of the P1-N1-P2 complex. An increase in latencies and a decrease in amplitude were observed in the condition of Delta-t 64 ms when compared to the condition without masking. There was a significant difference between the groups for the P1 component latency, in the condition without masking and Delta-t 64 ms, and for the N1 component latency, in the Delta-t 64 ms condition. It was verified that the P1 component latency in the condition without masking was significantly lower in the young adult group and the P1 and N1 component latency in the Delta-t 64 ms condition was higher in the elderly group. An increase in the latency of all components was observed in both conditions, mainly in the condition of Delta-t 64 ms, as a function of age.

**Table 2:** Description of intra-subject and between-group latencies.

Latencies (ms)	Young adult group	Adult group	Elderly group	*p* Value
	Mean ± SD	Mean ± SD	Mean ± SD	
	Median (P25; P75)	Median (P25; P75)	Median (P25; P75)	
P1 (without masking)	36.05 ± 0.03	54.40 ± 8.59	55.00 ± 11.34	*p*^d^ < 0.001^a^, ^b^
	36.05 (36.02; 36.07)	52.50 (49.00; 57.75)	53.50 (44.75; 65.00)	
P1 (Delta-t 64 ms)	272.30 ± 55.71	272.80 ± 25.56	323.70 ± 49.27	*p*^f^ = 0.025^b^, ^c^
	260.00 (236.25; 302.25)	273.00 (246.75; 302.50)	327.00 (269.00; 365.75)	
*p* Value	*p*^(4)^ < 0.001^g^	*p*^(4)^ < 0.001^g^	*p*^(4)^ < 0.001^g^	
N1 (without masking)	96.70 ± 10.48	99.90 ± 14.91	106.30 ± 20.22	*p*^e^ = 0.598
	98.00 (86.75; 104.00)	98.50 (86.50; 109.50)	98.50 (91.00; 118.00)	
N1 (Delta-t 64 ms)	321.90 ± 61.05	326.80 ± 33.08	371.30 ± 35.12	*p*^e^ = 0.039^b^, ^c^
	325.00 (275.50; 357.00)	326.50 (305.75; 353.75)	357.50 (343.50; 403.50)	
*p* Value	*p*^(4)^ < 0.001^g^	*p*^(4)^ < 0.001^g^	*p^(^*^4)^ < 0.001^g^	
P2 (without masking)	167.40 ± 14.25	181.50 ± 29.01	176.40 ± 22.66	*p*^d^ = 0.388
	173.50 (150.75; 179.00)	174.00 (169.25; 200.50)	181.00 (152.75; 193.50)	
P2 (Delta-t 64 ms)	385.50 ± 69.69	398.80 ± 31.96	436.70 ± 38.66	*p*^d^ = 0.074
	391.00 (326.00; 426.50)	399.50 (373.00; 427.50)	449.50 (405.75; 464.00)	
*p* Value	*p*^(4)^ < 0.001^g^	*p*^(4)^ < 0.001^g^	*p*^(4)^ < 0.001^g^	

ms, milliseconds; SD, standard deviation.

a Statistically significant difference of the means between the young adult and adult groups.

b Statistically significant difference of the means between the young adult and elderly groups.

c Statistically significant difference of the means between the adult and elderly groups.

d F-Test (ANOVA) with Tamhane’s multiple comparisons.

e Kruskal Wallis test with Conover’s all-pairs comparison.

f F-Test (ANOVA) with Tukey’s multiple comparisons.

g Significant difference at the 5.0% level.

**Table 3:** Description of intra-subject and between-group amplitudes.

Amplitude (µV)	Young adult group	Adult group	Elderly group	*p* Value
	Mean ± SD	Mean ± SD	Mean ± SD	
	Median (P25; P75)	Median (P25; P75)	Median (P25; P75)	
P1 (without masking)	4.78 ± 2.69	6.38 ± 2.72	5.85 ± 2.08	*p*^a^ = 0.312
	4.38 (2.99; 5.64)	5.17 (4.42; 7.91)	5.36 (4.12; 7.81)	
AP1 (Delta-t 64 ms)	3.69 ± 1.13	3.74 ± 1.62	3.23 ± 2.30	*p*^a^ = 0.520
	3.76 (2.63; 4.73)	3.65 (2.64; 4.84)	2.14 (1.71; 5.00)	
*p* Value	*p*^(5)^ = 0.275	*p*^(4)^ = 0.022^c^	*p*^(4)^ = 0.028^c^	
N1 (without masking)	8.22 ± 4.12	9.77 ± 3.73	9.70 ± 2.63	*p*^b^ = 0.551
	7.66 (5.06; 12.11)	8.67 (6.61; 14.00)	10.12 (7.44; 12.03)	
N1 (Delta-t 64 ms)	4.11 ± 2.45	4.23 ± 1.92	3.13 ± 1.07	*p*^a^ = 0.348
	3.69 (2.48; 4.67)	3.83 (3.20; 4.89)	2.95 (2.40; 3.93)	
*p* Value	*p*^(4)^ = 0.007^c^	*p*^(4)^ = 0.001^c^	*p*^(4)^ < 0.001^c^	
P2 (without masking)	5.79 ± 3.12	5.23 ± 3.15	6.89 ± 2.77	*p*^a^ = 0.221
	4.55 (3.56; 8.83)	4.15 (3.26; 7.02)	6.09 (4.48; 9.80)	
P2 (Delta-t 64 ms)	3.68 ± 2.47	2.97 ± 2.25	2.83 ± 1.13	*p*^a^ = 0.658
	2.96 (1.80; 4.99)	2.08 (1.44; 4.08)	2.81 (2.05; 3.23)	
*p* Value	*p*^(4)^ = 0.009^c^	*p*^(4)^ = 0.043^c^	*p*^(4)^ = 0.003^c^	

µV, microvolts; SD, standard deviation.

a Kruskal Wallis Test with Conover’s all-pairs comparison.

b F-Test (ANOVA) with Tamhane’s multiple comparisons.

c Significant difference at the 5.0% level.

The variability expressed through the standard deviation is reasonably high in the variables: amplitude of the P1 component of young adults in the condition without masking; amplitude of the N1 component of young adults in both conditions; amplitude of the P2 component of young adults and middle-aged individuals in both conditions.

**Figure 1:**
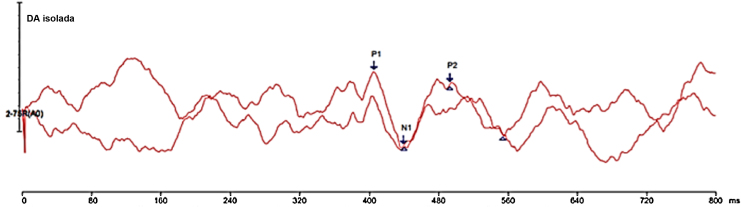
Demonstration of elicited responses without masking.

**Figure 2:**
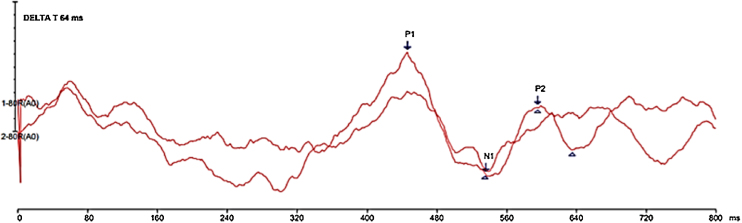
Demonstration of the elicited responses in the Delta-t 64 ms condition.

Figure 1, Figure 2 show the responses elicited under the two conditions of stimulus presentation, with the two reproductions of the tracing and the indication of the P1-N1-P2 complex.

## Discussion

The senescence of the auditory system may explain the fact that the elderly group, even with thresholds considered to be within the normal range,^7^ have higher hearing thresholds than the other two analyzed groups.^15^

Considering a previous study of behavioral audiological assessment,^5^ it is possible to observe that the difficulty in discriminating the target sound in the presence of noise may be present even when the peripheral hearing of the elderly individual is within the normal range.^7^ These elderly people have difficulty in discriminating the target sound even when the presented noise is characterized by being modulated, which facilitates the capture of acoustic cues.^5^ Based on the analysis of the latencies of the P1-N1-P2 complex in this study (Table 2), it is possible to observe an agreement between the electrophysiological assessment and the behavioral assessment, showing that the elderly are more influenced by noise than younger adult listeners.

Higher latencies indicate that the arrival of the electrical stimulus to the auditory cortex took longer and, in this case, the presentation of noise before the target sound in the Delta-t 64 ms condition directly influences latencies. This result was already expected and, therefore, the comparison of latencies between the two stimulus presentation conditions is irrelevant for the study of the forward masking phenomenon. The reduced amplitudes in the Delta-t 64 ms condition demonstrate that with the presentation of noise, the cortical responses are expressed at a lower magnitude, characterizing the forward masking phenomenon^4^, ^16^ (Table 2).

The components that showed more significant differences between the groups in their latencies were the P1 components in the two presentation conditions and the N1 component in the Delta-t 64 ms condition. These two initial CAEP components have the primary auditory cortex as possible generators, with the N1 component being an important marker of auditory cortical activity related to auditory decoding and discrimination.^16^

With advancing age, individuals become less and less able to regulate afferent sensory information and are more sensitive to disturbing information.^17^ Older listeners are more easily distracted by irrelevant stimuli, as they take longer to overcome previously presented sounds, contributing to impaired speech understanding.^16^

Confirming this hypothesis, the results of the present study showed that the latency of the P1 component was considerably lower in the group of young adults than in the other groups, even when the evaluated condition was the one without masking, that is, without noise presentation. The hypothesis is even more confirmed due to the fact that the group that was most affected by the increase in the P1 and N1 component latencies in the Delta-t 64 ms condition was the elderly group, thus showing that this group suffers the most influence of forward masking phenomenon on electrophysiological results.

Moreover, Table 2 shows that there is an increasing difference in latencies as a function of age, that is, it may be that cortical responses related to latencies tend to be directly proportional to age.^16^ Therefore, these data becomes relevant for the creation of parameters and biological marks of normality by age group for the CAEPs.

The high standard deviation observed in the results may be related to the sample size, that is, if the sample of study participants were larger, the results could be more homogeneous; therefore, it is suggested to continue this study.

Based on the Brainstem Auditory Evoked Potential exam with speech stimulation, it is possible to observe the effect of forward masking. Furthermore, it is possible to describe that the increase in forward masking phenomenon can be considered inversely proportional to the distance between the noise presentation and the speech stimulus.^18^ With the results obtained in this study, it is possible to consider that the electrophysiological tests, from the analysis of brainstem potentials to cortical potentials, are becoming efficient for the analysis of forward masking phenomenon as a function of age, being more sensitive than the behavioral tests.^19^, ^20^

The CAEPs are electrophysiological responses related to a test that allows the assessment of central auditory function, capturing the bioelectrical activities of the auditory pathways, allowing the measurement of auditory information processing in an objective and non-invasive way.^10^ The speech stimulus in this exam provokes exogenous responses, which are represented by the P1-N1-P2 complex.^16^ The clinical application of the CAEPs is still not prevalent in the field of audiology; however, the test has shown to be relevant, including for the study of speech comprehension in the presence of noise.^4^, ^21^, ^22^

One of the limitations of the present study was the difficulty in indicating the P1-N1-P2 complex, since, in the 64 ms Delta-t condition, there seems to be the presence of two complexes. More forward masking phenomenon studies, with a larger sample of individuals, are required for this understanding, thus making it possible to standardize CAEP measurements, so that this exam finds a clinical use that can complement the audiological assessment.

## Conclusion

It was possible to observe the forward masking phenomenon in the electrophysiological measurements (P1-N1-P2 complex) of the CAEP in young adults, adults and elderly people with normal hearing, with latency and amplitude measurements varying according to the condition of stimulus presentation and age group.

The presentation of noise prior to the target sound increases latency and reduces the amplitude of the CAEP components, suggesting the presence of forward masking phenomenon especially in the elderly. It is suggested that the elderly are more influenced by noise in the CAEP responses.

## Funding

This study received financial support from the Coordination for the Improvement of Higher Education Personnel – Brazil (10.13039/501100002322CAPES) – funding code 001.

## Conflicts of interest

The authors declare no conflicts of interest.
